# Clinic of Professor Dunglison

**Published:** 1844-12-14

**Authors:** Samuel G. White


᠎# THE MEDICAL EXAMINER,

### And Record of Medical Science.

Vol. VII.] PHILADELPHIA, SATURDAY, DECEMBER 14, 1844. [No. 25.

### CLINICAL LECTURES AND REPORTS.

### PHILADELPHIA HOSPITAL.

CLINIC OF PROFESSOR DUNGLISON.

*November* 23, 1844.

(Reported by Mr. Samuel G. White.)

[☞ In the report of the last clinic, p. 278, col. 2, line 19, for *left* read *right.*—S. G. W.]

Professor Dunglison commenced by remarking, that there is at present in the wards a well marked case of

SCARLATINA, to which he was desirous of directing the attention of the class, but inasmuch as it might be imprudent to introduce the patient for fear of its being communicable by contagion—although he is not wholly convinced that the disease is contagious—he would merely indicate the mode of treatment which he is pursuing.

The patient, Jane C., æt. 13, entered the house on the 21st inst., with the ordinary symptoms of the anginose variety, in the second degree of the eruption, at which time she was seen by the lecturer. Thus far the case has proceeded favourably. Since her entrance, the treatment has consisted essentially in sponging the surface with a cool dilute solution of chlorinated soda, (liq. sodæ chlorinatæ f.℥ij., aquæ Oj.—M.) gargling the throat with a mixture of muriatic acid, thirty drops to six fluid ounces of water, three or four times daily; the administration of small doses of ol. ricini and the use of ice internally. In simple cases of scarlatina but little treatment is necessary. It is very important in this, as in analogous affections, to open the bowels daily by some gentle means, for there is a tendency in the morbid action to extend itself from the skin to its prolongation inwards, constituting the mucous membranes, and unless the vitiated secretions, thus produced, are evacuated, their collection may prove an additional source of irritation, and the symptoms be aggravated. It is, in fact, from the extension inwards of this and other exanthematous diseases, that serious complications arise, which are frequently the cause of death; and the mucous membranes of the respiratory and digestive apparatus are most liable to suffer, hence the frequent occurrence of diarrhœa and dysentery, and of bronchitis and pneumonia, which last, as will be seen hereafter, may perhaps be considered a bronchitis of the terminal air tubes.

As scarlatina is another example of disease, which may be regarded as essentially self-limited, our endeavours must be restricted to combating the morbid phenomena as they arise. In fulfilling these indications, the treatment just detailed may be pursued in similar cases with success, modifying it, of course, according to circumstances. It will be continued in the present instance, and the result be reported to the class at the next clinical lecture.

The Professor next exhibited the expectoration of the patient labouring under

HÆMOPTYSIS, to whose condition, as illustrating one form of pulmonary transudation, he directed the attention of the class at the last lecture. The sputa, which were exhibited, still contain a considerable amount of blood; and they are more nummular and puriform than before. Little benefit can be expected from any remedial agent in finally arresting the hæmoptysis in this case, inasmuch as in all probability, as stated in the last lecture, it is caused by ulceration of one or more of the pulmonary vessels, and the treatment, therefore, will still be palliative. In consequence of an increase of febrile excitement, the solution of iodide of iron, which she was using, will be superseded by a more cooling astringent, as the dilute sulphuric acid, associated with the tincture of digitalis.

To exhibit the progress of the case the patient with

TYPHOID FEVER

was then introduced. The gurgling in the right iliac fossa, with slight diarrhœa, continued, but the *taches rouges* have entirely disappeared; the tongue and skin have become moist; the pulse is slower; the intellectual faculties are improved, and in fact the case is proceeding most favourably. Although she has been perspiring freely, no sudamina are perceptible. The progress of the case, thus far, fully confirms the statements made in the last lecture, that the mildest means only are indicated for its treatment, the more prominent symptoms being combated as they may arise. None such, however, have supervened.

The use of ice is now no longer necessary, as the skin is in a perspirable condition, and the tongue moist. It will be sufficient to give some slightly refrigerant mixture—the effervescing, for example—to restrict the diet, and keep the bowels gently evacuated. Should it become necessary, later on in the disease, general and mild excitants may be cautiously resorted to. Any violent, irritating plan of treatment, adopted with the view of arresting it, would be useless, and positively injurious. The lecturer confidently looks for a favourable termination in this case, and will report its progress.

A case of

RENAL DROPSY

next occupied the attention of the class. The lecturer observed, that he had been fortunate in being able to present already two cases of this affection, whereas during the last entire course he was unable to exhibit a single case.

The class would remember, that in the introductory lecture to the clinical course some observations were made on the value of attending to the secretions, especially the renal, as a means of studying disease, and that the indications derived from it, in certain dropsical affections, are of much importance. The urine exhibited on that occasion, as containing albumen in considerable quantity, was voided by the patient now before them.

Sarah C., æt. 44, entered the hospital under symptoms of effusion into the thoracic and abdominal cavities, and of anasarca. The feet and legs were much swollen at the time, and they have increased since. On examination, the urine was found to contain a large proportion of albumen. She had received the usual treatment for dropsy, but experienced only temporary relief; the hydropic accumulation still persisting to a varying extent. In this case, the lecturer observed, we have another example of transudation, but instead of blood, as in the patients whose cases were described in the last lecture, it consists of the watery portions of that fluid. All the local phenomena of dropsy are present, the part is swollen and pits on pressure. It is what may be termed hydropic, in contra-distinction to other transudations.

It was before remarked, that dropsical effusions may be dependent either on a loss of balance between the exhalants and absorbents of a part, or on some mechanical impediment, or disturbance in circulation, dependent upon a morbid condition of certain of the abdominal viscera. That obstruction to, or disturbance in, the circulation may give rise to a transudation of the more fluid portions of the blood, is shown by the frequent occurrence of dropsy of the peritoneum, as a result of hepatic condensation or enlargement, whereby the blood is prevented from passing freely through the portal vessels,—as a result of enlargement of the spleen, as after repeated attacks of intermittent fever, and of morbid conditions of the heart and kidney. In the case before the class the dropsy is dependent upon disturbance of the circulation, produced by renal disease. The kidney, as is well known, consists of two distinct portions, one of these, the cortical, has for its functions the secretion of the urine; the other, the tubular, that of conducting the urine to the pelvis of the kidney. In the peculiar morbid state under consideration, there is an alteration in the structure of the cortical or secreting portion of the viscus, which assumes a granular appearance, and has hence been called "granular disease of the kidney," or "Morbus Brightii," after the individual who first drew attention to it. In consequence of this organic change in the great secreting organ the circulation is disturbed, and as a result of this, effusion takes place into the cellular tissue and into the serous cavities.

This case is one of general dropsy, for there is not only a collection of fluid in the peritoneal sac, but in the general cellular tissue. The abdomen, you may perceive, continued the Professor, is greatly distended, but it does not follow that this distension is produced entirely by liquid. If percussion, indeed, be made over the anterior part of the abdomen, while the patient lies on her back, the sound is resonant,—not flat as it would be if the cavity contained only liquid. This resonance is due to the presence of air in the intestines, which float on the surface of the water and being now interposed between it and the parietes of the abdomen, give rise to the tympanitic or clear sound just referred to. That this is the case, may be proved by continuing the percussion over the abdomen on both sides to the spine, when the sound rendered will be found to become flat when the abdomen is struck over the seat of the liquid; and if the course of the percussion be changed, the resonance will increase until we reach the portion occupied by the intestines only. Should doubt, however, still exist as to the presence of fluid, the patient may be made to sit up when the water will gravitate to the lower part of the abdominal cavity, where it may be detected by the flat sound on percussion, and the intestines will now occupy the upper portion of the abdomen and cause a clear sound to be rendered. Thus, by causing the water to change its position, we may acquire sure indications of its presence. But there is yet another mode of determining this by percussion. If the fingers of one hand be placed lightly in contact with the abdomen, and the opposite part be gently and quickly struck with the fingers of the other hand, a distinct sense of fluctuation may be perceived. In this case, as these signs are all present, we may unhesitatingly conclude that the patient has dropsy of the belly, or ascites.

[The lecturer here practised percussion over the abdomen, to establish the views he had expressed.]

In this and in almost every case, the dropsy must be considered merely as a symptom, the pathological cause of which is the important question to be determined. In addition to what has been termed the hydropic diathesis or tendency, there usually exists some cause, which, by producing disturbance or irregularity of the circulation, gives rise to the accumulation of the serous secretion. Disease of any of the solid viscera may thus lay the foundation for local hyperæmia, and if the hydropic diathesis exist there will be dropsical accumulation.

Unfortunately the remedies in these cases are not potent, and our indications of treatment are restricted. If there be active dropsy dependent upon too much action of the exhalants, blood-letting, by diminishing the amount of the circulating fluid, may promote absorption, and the fluid may be thus removed. It is in cases of this active character, that such marked benefit results from venesection, followed by other means that diminish the amount of the circulating fluid, such as diuretics, hydragogue cathartics, &c. But in the cases of dropsy that generally occur in eleemosynary institutions, the hydropic tendency is so marked, and the patients of such broken down habits, that venesection cannot often be resorted to, and therefore other agents must be sought for. Generally, diuretics and cathartics are selected. These articles act both as revellents on the intestinal surface, and diminish the amount of the watery portions of the blood, which, as before stated, tends to increase the energy of absorption.

Jalap combined with the bitartrate of potassa; the pulvis jalapæ comp., of the Edinburgh Pharmacopœia is much employed as a cathartic in dropsy. It produces copious watery evacuations, and the bitartrate is possessed at the same time of diuretic properties. Often, however, in consequence of morbid states of the intestinal tube, our remedies cannot be directed to that surface, and we are compelled to seek some other. By common consent, the kidney is chosen, and diuretics are substituted for cathartics. Indeed, they must be regarded as the main agents prescribed in dropsy. But here again a difficulty arises in certain cases as in the present. The kidney also is in a diseased, some think inflammatory, condition, which is probably the cause of the dropsy; and the question arises how far excitants can be safely employed, which act on that viscus?

In such cases, it is probable, that mild renal excitants will not be injurious, and hence it is customary to administer an infusion of juniper berries, or a weak solution of bitartrate of potassa, singly or combined, as a common drink. With more propriety, perhaps, resort is had to small doses of squill and digitalis, which prescription the patient before the class is now taking. These and similar articles excite the secretion of the kidney, without stimulating the organ too much, and are often of decided benefit. At present, the patient is using the following pill:

℞. Pulv. scillæ
—— digital. *aa.* gr. i.
Ext. taraxaci, q. s. ut fiat pilula.

One of these to be taken three times a day. The pill has increased the flow of urine and lessened the force of the circulation, but it cannot be expected to effect any change in the organic lesion that causes the effusion; and so long as the cause remains, the effects will continue. The treatment mentioned will be pursued, and its results reported at some future time. No benefit, however, can be expected from any remedy.

As the physiology of the alimentary canal, had engaged the attention of the class during the week in the Jefferson Medical College, the Professor, in pursuance with his plan, as noticed in the previous lecture, presented sundry cases elucidative of its pathology.

The first of these was a person who had been severely affected with

MERCURIAL STOMATITIS.

She had suffered under an attack of typhoid pneumonia, which resisted the ordinary modes of treatment; and it was therefore deemed necessary to place her under the revellent influence of mercury, with the view of breaking in upon the morbid chain; notwithstanding every care, however, the mercurial affection had gone too far; but it had apparently produced the most salutary effect on the disease for which it was prescribed. The Professor here took occasion to observe, that in cases of pneumonia, which had assumed the chronic or typhoid character, he had found mercury, carried so far as merely to effect the mouth, very beneficial.

These cases of mercurial stomatitis are mostly self-limited, or incapable of being wholly arrested, although they may be palliated by treatment. The symptoms that characterize it are—peculiar fœtor of the breath, falling away of the gums from the teeth,—the gums being topped by a whitish mucous matter. There may also be an increased flow of saliva, and there is generally more or less constitutional irritation—"mercurial fever." The action of the drug may stop here; but at times, though not so often as formerly, the morbid action proceeds, profuse salivation ensues and instead of simple inflammation, sloughing attacks the soft parts about the mouth, which may result in their destruction, and may even extend so far as to destroy the whole cheek, and even the bones of the face. These ravages produced by the excessive use of mercury were at one time very frequently seen, when it was the practice to continue it until the patient secreted a quantity of saliva measured by the intensity or duration of the disease.

The lecturer stated, that some years since he attended a lady of much respectability, whose mouth was almost closed in consequence of contraction produced by ulcers thus arising, but fortunately, by the aid of an appropriate instrument, she was enabled, by extending the firm tissue of the cicatrices, to separate the jaws to a considerable extent, and was thus relieved from the inconvenience of receiving nourishment only through an aperture between the teeth, as she had been compelled to do for some time.

A case of a more aggravated character, and of much interest, had been recently exhibited to the clinical class by his friend Professor Pancoast.

There is great difference in individuals as to their susceptibility, in regard to the peculiar action of mercury, and the lecturer had noticed in the wards of the hospital, that they appeared to be more readily affected than in private practice.

The patient, under consideration, is now recovering from the stomatitis. She has been purged freely by sulphate of magnesia, and she uses a mouth wash of chlorinated soda. In all cases of this description, disinfecting substances are useful,—not only by correcting the fœtor, but being generally of an excitant or astringent character, they are calculated to induce a new action in the ulcerated surfaces and promote their cicatrization. For this purpose, the various chlorinated preparations, kreasote, &c., are usually resorted to. The lecturer remarked, that he had found the simple sulphate of alumina—not the sulphate of alumina and potassa—to possess disinfecting powers superior to any other article he had employed,—destroying almost immediately the fœtor of the most offensive animal substances. He employs it in the form of solution, and in cases of fœtid ulcers it will no doubt be found exceedingly advantageous, as it combines with the other properties, that of astringency, which renders it peculiarly applicable to many such cases. It had been prescribed in the surgical wards of the hospital and with success as a disinfectant and detergent to foul ulcers. The solution will be directed, if necessary, in the present case, and an opportunity may be afforded to the class to form some idea of its efficacy in congenerous affections. Every form of astringent,—alum, acetate of lead, sulphate of copper, tincture of chloride of iron, and nitrate of silver have been prescribed as topical remedies. But, although they may be of some benefit as palliatives, they will generally disappoint expectation, for the disease. as before stated, often continues, apparently uninfluenced by them.

The next patient presented before the class was affected with

PHARYNGITIS

with ulceration and relaxation of the fauces, which from their long resistance to treatment, would appear to be dependent upon some *vice* in the system, in all probability not venereal—but strumous. The parts about the throat, velum palati and uvula are in a lax condition: the uvula especially is very pendulous, and may require excision. On pressing on the larynx, the patient complains of pain, which is probably caused by the same condition of the lining membrane of the larynx; and as there is some cough, an affection of the upper portion of the lung may exist, as is very liable to happen in such cases. This will be examined into before the class meets again—the state of profuse perspiration in which he now is, rendering it unsafe to expose his chest at this time. The treatment under which the throat has improved, and which usually is very efficient has consisted in touching the inflamed and ulcerated parts, with a mop dipped in a solution of nitrate of silver—(*Argent. nitrat.* gr. xx. *Aq. destil:* f.℥j.) In addition to this, attention has been directed to the constitutional condition; and he has been put upon the free use of the iodide of potassium.

The lecturer now introduced another patient, who presented a very emaciated appearance, the consequence of

ULCERATION OF THE INTESTINES, which had continued since the first of September last. According to the patient's own account, the attack began with vomiting, and this was succeeded by numerous alvine evacuations, sometimes amounting to 20 or 24 during the day, and containing blood mixed with slimy matter; he did not however confine himself to bed. At first, there was considerable tenderness over the umbilical region, indicating that the small intestine was probably involved in the disease, but this tenderness has disappeared. There was, also, tenesmus, with pain in the back, and uneasiness after discharges, showing that the rectum was probably implicated. From the continuance and character of the affection, the lecturer had every reason to believe that ulceration was present. Dr. Stokes was the first, he believed, to direct attention to the fact, that in many cases of inveterate dysentery there are ulcerations within the rectum, and that by applying astringents and other agents to them, a cure might be effected. When these ulcers, however, are situate high up in the intestines, no agent can be brought to act directly on the diseased surface, and hence such cases are extremely difficult to treat. It is customary, however, to administer certain of the mineral astringents in small doses, but it can be easily comprehended, that as they must undergo admixture with the aliments, and with the various secretions from the supra-diaphragmatic portion of the digestive canal, and of the stomach and intestines, their active properties must be much diminished if not destroyed, before they reach the seat of the disease: and it is more reasonable to suppose that, in such cases, their action is confined to the revellent and tonic impression which they make on the stomach, and does not consist in any direct impression on the ulcers themselves.

In the present instance, the lecturer, from the functional phenomena, suspected that ulceration existed low down in the intestines, and on making an examination, by means of the speculum, the resident physician, in charge of the wards, perceived several ulcerated spots just within the internal sphincter. The patient has much improved lately under the use of an astringent solution, consisting of four grains of the sulphate of copper to one ounce of water, thrown up in injection: the number of evacuations now being only three during the day, but if this improvement does not continue, the solid nitrate of silver, as recommended by Dr. Stokes, will be applied immediately to the surface of the ulcers. Occasionally much benefit is found to result from counter-irritation over the abdomen, or the revellent effect of mercury, produced by rubbing the mercurial ointment over the abdomen, so as to slightly affect the mouth.

In conclusion, the professor exhibited the results of a case that died from

DROPSY, more as a pathological curiosity than to establish any point of diagnosis. In removing the liver of a female, the kidney of one side was brought away, adhering firmly to it, whilst that of the opposite side was so displaced as to be seated transversely, and in contact with the diaphragm, instead of longitudinally, as it naturally is, in the abdomen. These displacements may have been congenital, or they may have been caused by the pressure of the gravid uterus in pregnancy, which forced one kidney upwards in contact with the diaphragm, and the other against the liver, to which it became adherent by inflammation. The cause of the dropsical effusion was not apparent from the necroscopy: there was, however, a granular appearance of the kidneys and indistinctness in the two portions—the cortical and tubular; but this was very slight, and if any renal disease existed, it was manifestly in its incipiency. The liver of this person also served well to exhibit the difference between that viscus in the more healthy and in the diseased state. Although the patient had been very intemperate, that change in the nutrition of the viscus had not been caused, which intemperance in the use of ardent spirits usually produces, and which it had done in a marked manner in the liver of another patient who died of phthisis, and who was very intemperate, which the lecturer then placed alongside the healthy one: the difference between the two was most marked,—the latter being "fatty," and manifestly "*cirrhosed.*" The other viscera of this patient appeared healthy, with, perhaps, the exception of the spleen, which was very much atrophied. The use of this viscus, in the animal economy is not well understood. It certainly is not of vital importance, as it has, on repeated occasions, been removed without any serious consequences, when it protruded in wounds of that region. In the lower animals, it has often been taken out experimentally. Dupuytren removed it from forty dogs, twenty of which died from peritoneal inflammation, but the remainder seemed to suffer no inconvenience. On the other hand, some of them grew fat. In malarious districts, there not unfrequently exists, what has been termed, in India, "splenic cachexia," in which the spleen is greatly enlarged, and there is a strong tendency to hemorrhages from various organs, as the stomach, bowels, &c. It is possible, that the spleen may serve, under particular conditions of the system, as a diverticulum for the blood, and this view seems to receive support from the engorgements of the viscus during long continued intermittents, in the cold stage of which the blood leaves the circumference and is collected in the internal organs. As the spleen can be entirely removed without danger, it can be understood, that its atrophy need not produce any serious effect: it is, in fact, occasionally found very much diminished; in one case seen by the lecturer, it was no larger than a nutmeg, and yet the patient did not seem to suffer.

